# Cytokine Balance in Human Malaria: Does *Plasmodium vivax* Elicit More Inflammatory Responses than *Plasmodium falciparum*?

**DOI:** 10.1371/journal.pone.0044394

**Published:** 2012-09-04

**Authors:** Raquel M. Gonçalves, Kézia K. G. Scopel, Melissa S. Bastos, Marcelo U. Ferreira

**Affiliations:** 1 Department of Parasitology, Institute of Biomedical Sciences, University of São Paulo, São Paulo, Brazil; 2 Department of Parasitology, Microbiology and Immunology, Institute of Biological Sciences, Federal University of Juiz de Fora, Juiz de Fora, Brazil; Université Pierre et Marie Curie, France

## Abstract

**Background:**

The mechanisms by which humans regulate pro- and anti-inflammatory responses on exposure to different malaria parasites remains unclear. Although *Plasmodium vivax* usually causes a relatively benign disease, this parasite has been suggested to elicit more host inflammation per parasitized red blood cell than *P. falciparum.*

**Methodology/Principal Findings:**

We measured plasma concentrations of seven cytokines and two soluble tumor necrosis factor (TNF)-α receptors, and evaluated clinical and laboratory outcomes, in Brazilians with acute uncomplicated infections with *P. vivax* (n = 85), *P. falciparum* (n = 30), or both species (n = 12), and in 45 asymptomatic carriers of low-density *P. vivax* infection. Symptomatic vivax malaria patients, compared to those infected with *P. falciparum* or both species, had more intense paroxysms, but they had no clear association with a pro-inflammatory imbalance. To the contrary, these patients had higher levels of the regulatory cytokine interleukin (IL)-10, which correlated positively with parasite density, and elevated IL-10/TNF-α, IL-10/interferon (IFN)-γ, IL-10/IL-6 and sTNFRII/TNF-α ratios, compared to falciparum or mixed-species malaria patient groups. Vivax malaria patients had the highest levels of circulating soluble TNF-α receptor sTNFRII. Levels of regulatory cytokines returned to normal values 28 days after *P. vivax* clearance following chemotherapy. Finally, asymptomatic carriers of low *P. vivax* parasitemias had substantially lower levels of both inflammatory and regulatory cytokines than did patients with clinical malaria due to either species.

**Conclusions:**

Controlling fast-multiplying *P. falciparum* blood stages requires a strong inflammatory response to prevent fulminant infections, while reducing inflammation-related tissue damage with early regulatory cytokine responses may be a more cost-effective strategy in infections with the less virulent *P. vivax* parasite. The early induction of regulatory cytokines may be a critical mechanism protecting vivax malaria patients from severe clinical complications.

## Introduction

Malaria is the most devastating protozoan disease afflicting humans. Every year, *Plasmodium falciparum* causes around 0.5–3 million deaths, mostly in sub-Saharan Africa [1], while *P. vivax,* the most widespread human malaria parasite [2], causes 130–390 million clinical episodes [3]. Although the pathophysiology of vivax malaria remains poorly understood [4], adherence to endothelial cells [5] and sequestration of infected red blood cells in deep vasculature [6] have been recently demonstrated, while severe vivax malaria is becoming increasingly common in South and Southeast Asia, Oceania and South America [7], [8].

Clearing malaria parasites without inducing major host pathology requires a finely tuned balance between inflammatory and regulatory cytokine responses, whose timing and magnitude is crucial in determining malaria patient outcome [9]. Early production of tumor necrosis factor (TNF)-α, interferon (IFN)-γ, interleukin (IL)-6, IL-12 and other inflammatory cytokines allows fast *P. falciparum* clearance [10]–[12]. Once parasitemia is under control, regulatory cytokines such as IL-10 and transforming growth factor (TGF)-β are required to reduce the risk of severe disease [13]–[15].

Cytokine responses have been extensively described in *P. falciparum* infections in Africa and Oceania [12], [15], [16], but the mechanisms by which semi-immune subjects regulate their immune responses under low-level exposure to different malaria parasite species remain obscure. Plasma concentrations of the soluble TNF-α receptors sTNFRI and sTNFRII, which bind to circulating TNF-α and regulate its activity, correlate positively with parasitemia and disease severity in *P. falciparum* malaria in Africa [17]–[19], but scant data are available for *P. vivax* infections [20]. Although it has been suggested that *P. vivax* elicits greater host inflammation per parasitized red blood cell than *P. falciparum* [21], [22], we recently found a bias towards regulatory cytokines in uncomplicated vivax malaria in Brazil [23]. It remains unclear whether such a bias translates into milder clinical manifestations and decreased risk of severe disease [24].

The present study investigated the cytokine interplay in relation to the outcome of human malaria caused by *P. vivax*, *P. falciparum* or both species. Cytokine responses and levels of soluble TNF-α receptors were compared among malaria patients, asymptomatic carriers of malaria parasites and apparently healthy controls exposed to low-level malaria transmission in Brazil. To examine whether cytokine responses correlated with parasitological and clinical outcomes in *P. vivax* infections, parasitemias were estimated by real-time polymerase chain reaction (PCR) and the intensity of malaria-associated illness was quantified.

## Methods

### Study Participants

We recruited three groups of subjects exposed to hypoendemic malaria transmission in northwestern Brazil: (a) symptomatic malaria patients infected with *P. vivax, P. falciparum* or both species, (b) asymptomatic carriers of low-density *P. vivax* infection, and (c) healthy, non-infected controls. The symptomatic malaria patients (127 patients aged 12–78 years) were enrolled from malaria clinics in Plácido de Castro (Acre State) and Remansinho (Amazonas State) [25], [26]. They were infected with *P. vivax* (n = 85), *P. falciparum* (n = 30), or both species (n = 12), and had a clinical spectrum ranging from very mild illness to full-blown paroxysms but included no severe or complicated malaria cases. Clinical and laboratory data from 75 of these subjects had been previously reported [23] and here are combined with additional laboratory results from these same subjects and a complete analysis of an additional 52 subjects living in the same areas. All patients were treated free of charge in accordance with the current malaria therapy guidelines in Brazil (see File S1: Methods online). Paired blood samples were also collected from 39 *P. vivax-*infected patients around 28 (range, 26–30) days after being started on antimalarial chemotherapy, in order to evaluate cytokine levels during convalescence. Levels of some cytokines in 22 of these paired samples had been reported previously [23] and were combined here with those for 17 additional pairs.

Samples from asymptomatic carriers of *P. vivax* infection were collected from 45 subjects aged 4–56 years participating in an ongoing prospective cohort study of malaria risk factors in the farming settlement of Remansinho (Amazonas) [27]. These subjects had very low *P. vivax* parasitemias detected by real-time PCR, but were negative by thick smear microscopy; none of them had fever or any malaria-related symptom up to seven days prior to blood collection. Twenty apparently healthy subjects (aged 16–69 years) living in the same areas, who were negative for malaria parasites on both microscopy and real-time PCR, and had no slide-confirmed malaria episode in the past six months, served as malaria-exposed, non-infected controls. Demographic and clinical characteristics of the study subjects are given in Table 1. This study was approved by the Research Ethics Review Board of the Institute of Biomedical Sciences of the University of São Paulo, Brazil (792/CEP). Written informed consent was obtained from all study participants or their parents/guardians.

**Table 1:** Demographic, hematologic and clinical characteristics of study participants.

Characteristic	Non-infected controls	Symptomatic *P. vivax* infection	Asymptomatic *P. vivax* infection		Symptomatic *P. falciparum* infection	Symptomatic mixed-species infection	*P* value
Number of subjects	20	85		45*	30	12§	
Age (years)	32 (28–42)^a^	30 (21–43)^a^		23 (12–37.5)^b^	34 (26.5–46)^a^	33 (16–43)^ab^	0.012
Gender (% male)	55.5	62.4		48.9	66.7	54.5	0.538
Years of malaria exposure	28 (20–40)^a^	23 (15–35)^a^		14 (6–23)^b^	26 (20–33)^a^	22 (16–33)^ab^	0.0001
Hemoglobin level (g/l)	140 (118–152)	132 (120–143)		126 (116–144)	133 (122–142)	129 (116–139)	0.531
Anemia (%)	20.7	30.6		17.8	26.7	27.3	0.986
Platelets (10^9^/l)	-	158 (113–201)^a^		204 (166–242)^b^	202 (148–226)^b^	202 (147–266)^b^	0.002
Thrombocytopenia (%)	-	47.1^a^		8.9^b^	23.3^c^	27.3^ac^	0.006
Parasitemia (parasites/µl)	-	1184 (193–4594)^a^		16 (6–121)^b^	525 (130–4216)^ac^	228 (37–477)^c^	<0.0001
Geometric mean parasitemia	-	830.0		22.1	59.5	19.8	
Clinical index	-	0.9 (−1.5–3.3)^a^		-	0.3 (−1.5–3.0)^a^	−1.26 (−5.8–0.1)^b^	0.029
Duration of symptoms (days)	-	4 (2–5)		-	5 (3–9)	3 (1–5)	0.068

Clinical and laboratory data from 75 symptomatic malaria patients and apparently healthy controls had been previously reported [23] and here are combined with additional subjects living in the same areas. Data are expressed as median (interquartile range) unless stated otherwise and compared across three to five groups with Kruskal-Wallis tests (continuous variables) or χ^2^ tests (proportions); *P* values for this comparison across groups are presented in the right-hand column. Only when comparisons across groups showed statistical significance, further pairwise comparisons were made using Mann-Whitney tests (continuous variables) or χ^2^ tests (proportions). Values with different superscripts across a row indicate pairwise comparisons with statistical significance at the 5% level. The same value may have more than one superscript. When pairs of values across a row share one superscript (either the only or one of the superscripts associated with them), no statistically significant difference was found. Anemia was defined when hemoglobin concentration was below the following cut-off values: 120 g/l for adolescents aged 12–14 years and non-pregnant women, and 130 g/l for men aged ≥ 15 years. Thrombocytopenia was defined when the platelet count was below 150×10^9^/l.

* Number of subjects  = 27 for hemoglobin measurements and platelet counts.

§ Number of subjects  = 11 for hemoglobin measurements and platelet counts.

### Measurement of Clinical Symptom Intensity

All patients were interviewed using a structured questionnaire [28], [29], regardless of the infecting parasite species, in order to grade the intensity of 13 symptoms: fever, chills, sweating, headache, myalgia, arthralgia, abdominal pain, nausea, vomiting, dizziness, cough, dyspnea, and diarrhea. Clinical manifestations were considered to be absent, mild, moderate or severe and were assigned numerical scores of 0, 1, 2 or 3; fever was classified as absent, mild or severe (0, 1 or 2), and vomiting and abdominal pain were classified as either absent or present (0 or 1). To rank subjects according to the overall intensity of malaria-associated symptoms, numerical scores were aggregated into an index. We used principal component analysis (PCA), carried out using the XLSTAT software, version 7.5.2 (Addinsoft, New York, NY), to derive weights assigned to each symptom [30]. PCA is a multivariate statistical technique for summarizing the information contained in a set of variables to a smaller number of dimensions by creating a set of mutually orthogonal uncorrelated components (the principal components). The first principal component explains the largest possible variation in the data and can be used as a single value that captures the information from a set of variables into one composite measure. PCA was applied to variables (numerical scores associated with each symptom), normalized by its mean and standard deviation. The first principal component explained 27.7% of variability and gave the greatest weights to chills, fever, sweating, nausea, headache, and dizziness. The normalized scores assigned to each symptom were weighted using the PCA-derived factor loadings and summed to give a clinical index for each patient. The normalized scores were also used to build partial indexes that included only symptoms associated with paroxysms (fever, chills, and sweating) and body pains (myalgia, arthralgia, and abdominal pain).

### Clinical Laboratory Analysis

Blood cell counts and hemoglobin measurements were performed using an ABX Micros 60 automated cell counter (Horiba, Montpellier, France). Thick blood smears were stained with Giemsa and at least 100 fields were examined under 1000× magnification (corresponding to an average of 0.1 µl of blood) for malaria parasites by two experienced microscopists (see File S1, Methods online). A quantitative real-time PCR method that targets the 18S rRNA gene was standardized to estimate P. vivax and P. falciparum parasitemias (see File S1, Methods online). Parasitemia was expressed as the number of parasites per µl of blood; in subjects co-infected with P. vivax and P. falciparum, parasite counts for each species were summed to calculate total parasitemias.

### Cytokine and Soluble TNF-∝ Receptor Measurements

Plasma samples were examined for TNF-∝, IFN-γ, IL-4, IL-10, IL-12p40 and TGF-β using OptEIA capture ELISA kits (BD Biosciences, San José, CA), while levels of IL-6, sTNFRI and sTNFRII were measured using DuoSet capture ELISA kits (R&D Systems, Minneapolis, MN). Plasmas were tested in duplicate and concentrations were expressed as pg/ml. IL-12 is a heterodimer (known as IL-12p70) composed of two subunits, p35 and p40. The p40 subunit of IL-12 is usually produced in higher amounts than the bioactive p35 subunit and may act as a limiting factor for IL-12p70 secretion. In addition, IL-12p40 acts as an antagonist of human IL-12 receptor and inhibits IL-12-dependent immune functions. For these reasons, IL-12p40 in this paper is regarded as a regulatory or anti-inflammatory cytokine. Plasma levels of TNF-∝, IFN-γ, IL-4, IL-10, IL-12p40 and TGF-β for 75 acute-phase samples from symptomatic malaria patients and apparently healthy controls and for 22 convalescence samples had been previously reported [23] and here are combined with IL-6, sTNFRI and sTNFRII measurements in these same samples and a complete analysis in additional acute-phase and convalescence samples. To estimate the per-parasite production of cytokines and soluble TNF-α receptors [21], [22], measured concentrations (pg/ml) were divided by parasite counts estimated using real-time PCR (parasites/µl of blood); ratios are expressed as pg of cytokine per 10^3^ parasites (TGF-β, sTNFRI and sTNFRII) or per 10^6^ parasites (all other cytokines).

### Statistical Analysis

Cytokine levels with an overdispersed distribution were summarized as medians and interquartile ranges and compared across groups using nonparametric Kruskal-Wallis tests. When the Kruskal-Wallis test indicated a significant difference (*P*<0.05) among groups, pairwise Mann-Whitney *U* tests were carried out to determine where differences lay. Paired data (acute-phase vs. convalescence samples from the same patients) were compared using Wilcoxon signed ranks tests. Correlations were assessed using nonparametric Spearman rank correlation tests.

To determine whether independent variables (cytokine levels or ratios and sTNFR levels) correlated with any of the four outcome variables (hemoglobin levels, platelet counts, parasitemia, and clinical index), a series of multiple linear regression models were built. Given the relatively low number of subjects infected with other species, this analysis was limited to subjects infected with *P. vivax* for whom complete data were available. Parasitemias and cytokine levels and ratios were log_10_-transformed toward normality prior to analysis. Standardized regression coefficients (β) were interpreted to indicate the influence of a given predictor (independent variable) on each outcome (dependent variable), controlling for all other independent variables in the model. Separate regression models were built for each independent variable and each outcome. The first models included age (years), gender (female  = 0, male  = 1), and length of exposure to malaria (years) as independent variables. Parasitemia was included as an independent variable except when it was the outcome variable to be analyzed. To determine whether correlations between the outcome and particular cytokines (detected in partial models at 5% level of significance) were independent of each other, a backward stepwise approach was used to retain, in the final multiple linear regression model, only those variables that remained correlated with the outcome on the analysis after controlling for all other predictors. Analyses were performed using SPSS 16.0 software (SPSS, Chicago, IL), with statistical significance set at a 5% level.

## Results

### Characteristics of Study Subjects

Groups of symptomatic patients infected with *P. vivax, P. falciparum* or co-infected with both species were comparable to each other and to healthy control subjects in terms of age, gender, length of exposure to malaria transmission (years of residence in the Amazon), hemoglobin concentration, and anemia prevalence. Asymptomatic *P. vivax* carriers, however, were younger and had less cumulative exposure to malaria than the other study subjects (Table 1). Parasite counts were low to moderate (range, 2.6 to 118750 [median, 765] parasites/µl of blood) among symptomatic subjects and very low (range, 2 to 716 [median, 15.6] parasites/µl of blood) among those with asymptomatic infection. Among symptomatic patients, the highest and lowest geometric means were found in *P. vivax*-only and mixed-species malaria, respectively (*P* = 0.009 for this pairwise comparison, Mann-Whitney *U* test), consistent with lower parasite counts reported in mixed-species compared to single-species infections acquired in the same area [31]. Median platelet counts were lower, with higher prevalence of thrombocytopenia, in symptomatic *P. vivax* infections, compared to symptomatic *P. falciparum* and mixed-species infections, as well as to asymptomatic *P. vivax* infection.

### Clinical Expression of Malaria

**Figure 1:**
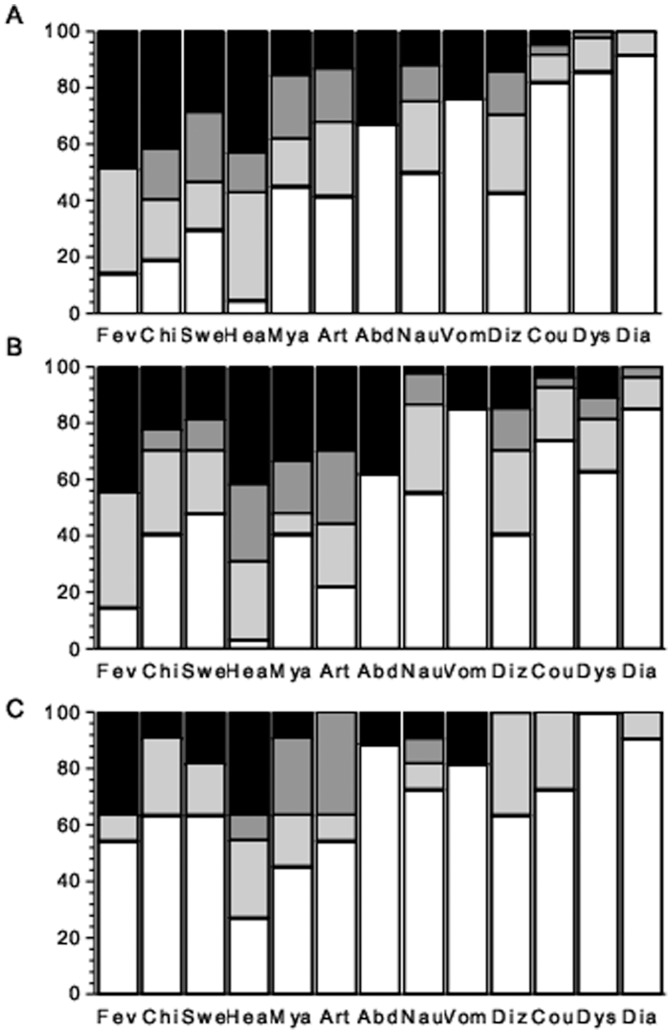
Severity of 13 malaria-associated symptoms in Amazonians with uncomplicated infection with *Plasmodium vivax* (A; n = 84), *P. falciparum* (B; n = 27) or both species (C; n = 11), diagnosed by thick-smear microscopy and confirmed by real-time PCR. Symptoms are abbreviated as follows: fever  =  Fev; chills  =  Chi; sweating  =  Swe; headache  =  Hea; myalgia  =  Mya; arthralgia  =  Art; abdominal pain  =  Abd; nausea  =  Nau; vomiting  =  Vom; dizziness  =  Diz; cough  =  Cou; dyspnea  =  Dys; diarrhea  =  Dia. Each bar segment represents the proportion of subjects reporting a given symptom as absent (no shading), mild (light gray), moderate (dark gray) or severe (black).

Clinical symptoms were analyzed for 84 laboratory-confirmed single-species symptomatic infections with *P. vivax*, 27 single-species infections with *P. falciparum*, and 11 mixed-species infections with *P. vivax* and *P. falciparum*. Headache (91.8%), fever (82.0%), chills (72.1%), sweating (63.1%), arthralgia (61.5%) and myalgia (55.7%) were the most prevalent individual symptoms (Figure 1), but typical paroxysm symptoms (fever, chills and sweating) coexisted in only 64 (50.8%) subjects. Significant differences across species were found, using sequentially Kruskal-Wallis and Mann-Whitney tests, in the intensity of chills (greater in vivax malaria than in falciparum malaria [*P* = 0.009] and mixed-species malaria [*P* = 0.001]), sweating (greater in vivax than in mixed-species malaria, *P* = 0.044) and dyspnea (greater in falciparum than in vivax malaria, *P* = 0.005) (Figure 1).

Differences in the overall clinical index suggest that mixed-species infections elicited milder symptoms, compared to single-species infections with *P. vivax* or *P. falciparum* (Table 1), consistent with intra-host between-species competition being beneficial to the host [31]. Opposite effects of mixed-species infections, however, have also been reported [32]. Paroxysms (quantified with partial index) were more intense in vivax malaria than in single-species *P. falciparum* and mixed-species infection (*P* = 0.028 and *P* = 0.003, respectively, Mann-Whitney *U* tests), while falciparum malaria patients had more intense body pain (myalgia, arthralgia, and abdominal pain) than vivax malaria patients (*P* = 0.041, Mann-Whitney *U* test).

### Cytokines and Soluble TNF-∝ Receptors during Malaria Infection

Plasma concentrations of IL-4, IL-12p40, IFN-γ, TNF-α, IL-10, sTNFRI and sTNFRII were higher in one or more groups of symptomatic patients, compared to non-infected controls (Table 2). Although vivax malaria was associated with more intense paroxysm-related symptoms, no pro-inflammatory imbalance was found in these patients. In fact, this group had greater concentrations of IL-10 (as described elsewhere [23], [33], [34]) and of sTNFRII than patients infected with *P. falciparum,* and higher levels of IL-10, IL-12p40, IFN-γ, sTNFRI and sTNFRII than patients infected with both species. Falciparum malaria was associated with higher levels of circulating plasma TNF-α than mixed-species infections.

**Table 2:** Absolute and relative levels of cytokines in study participants.

Cytokine	Non-infectedcontrols	Symptomatic *P. vivax* infection	Asymptomatic *P. vivax* infection	Symptomatic *P. falciparum* infection	Symptomatic mixed-species infection	*P* value
Number of subjects	18	81	45	30	12	
IL-4 (pg/ml)	1.9 (1.7–2.5)^a^	3.9 (2.9–6.0)^b^	4.7 (4.1–5.8)^c^	3.4 (2.7–4.9)^b^	3.0 (2.4–3.7)^b^	<0.0001
IL-4 per parasite(10^6^)	-	4 (1–29)^a^	334 (54–840)^b^	5 (1–41)^ac^	20 (5–89)^c^	<0.0001
IL-12 (pg/ml)	30.8 (28.4–50.7)^a^	63.8 (42.8–94.3)^b^	53.1 (34.1–82.3)^bc^	53.2 (36.4–71.8)^bc^	39.7 (27.1–63.6)^c^	0.033
IL-12 per parasite(10^6^)	-	53 (11–405)^a^	3814 (575–7809)^b^	84 (10–1023)^a^	154 (41–1118)^a^	<0.0001
IFN-γ (pg/ml)	5.4 (3.1–8.5)^a^	11.9 (5.5–36.8)^b^	4.0 (3.2–7.5)^a^	10.5 (4.4–61.3)^b^	3.8 (3.0–23.3)^a^	<0.0001
IFN-γ per parasite(10^6^)	-	12 (1–96)^a^	344 (64–829)^b^	20.9 (6–240)^a^	40 (12–343)^a^	<0.0001
IL-6 (pg/ml)	10.3 (9.5–17.9)	13.4 (10.0–22.4)	11.1 (10.4–13.3)	13.2 (9.8–16.6)	10.2 (9.8–11.4)	0.177
IL-6 per parasite(10^6^)	-	15 (4–64)^ac^	898 (129–2869)^b^	26 (5–119)^cd^	64 (19–263)^d^	<0.0001
TNF-α (pg/ml)	3.8 (3.0–4.3)^a^	6.8 (4.9–9.8)^bc^	3.6 (3.1–5.0)^a^	7.8 (6.1–10.8)^b^	4.7 (3.2–9.7)^ac^	<0.0001
TNF-α per parasite(10^6^)	-	7 (2–35)^a^	299 (42–793)^b^	20 (12–119)^ac^	31 (12–118)^c^	<0.0001
IL-10 (pg/ml)	6.2 (4.4–9.1)^a^	260.1 (53.5–532.7)^b^	10.1 (8.0–16.8)^c^	47.8 (19.7–298.5)^d^	24.8 (5.5–121.3)^cd^	<0.0001
IL-10 per parasite(10^6^)	-	137 (39–731)^a^	815 (206–1899)^b^	78 (39–304)^a^	147 (74–265)^a^	<0.0001
TGF-β (pg/ml)	379 (178–1440)^ab^	948 (415–3862)^c^	368 (192–970)^b^	826 (492–1936)^ac^	720 (487–3802)^c^	<0.0001
TGF-β per parasite(10^3^)	-	1 (0.1–8)^a^	29 (6–86)^b^	2 (0.2–7)^ac^	11 (1–73)^bc^	<0.0001
sTNFRI (pg/ml)	648 (474–1728)^ab^	3015 (1487–5622)^c^	1097 (870–1377)^a^	2481 (855–4966)^c^	1127 (641–3385)^ac^	<0.0001
sTNFRI per parasite(10^3^)	-	3 (1–11)^a^	62 (23–211)^b^	4 (1–12)^a^	11 (3–20)^a^	<0.0001
sTNFRII (pg/ml)	3259 (2540–8016)^a^	16060 (10442–32971)^b^	4128 (3030–6704)^a^	10217 (6760–20598)^c^	7659 (4881–20479)^c^	<0.0001
sTNFRII per parasite(10^3^)	-	14 (3–63)^ab^	247 (52–809)^c^	24 (4–97)^ad^	63 (12–212)^d^	<0.0001
IL-10/TNF-α ratio	1.6 (1.3–1.9)^a^	39.3 (6.5–70.5)^b^	3.0 (2.0–4.3)^c^	6.0 (1.5–33.4)^c^	3.2 (1.7–13.7)^c^	<0.0001
IL-10/IFN-γ ratio	1.4 (1.0–1.7)^a^	14.0 (3.2–39.3)^b^	2.5 (1.7–3.3)^c^	3.6 (1.5–9.7)^c^	3.5 (1.7–7.5)^c^	<0.0001
IL-10/IL-6 ratio	0.3 (0.2–0.5)^a^	9.6 (2.2–27.6)^b^	0.8 (0.6–1.5)^c^	3.9 (1.4–18.8)^b^	1.7 (0.5–9.6)^c^	<0.0001
sTNFRI/TNF-α ratio	619 (351–694)	405 (210–882)	281 (195–380)	306 (71.5–675)	188 (151–382)	0.066
sTNFRII/TNF-α ratio	2252 (1112–3639)^ab^	2902 (1284–5009)^a^	1004 (596–1766)^b^	1187 (539–2619)^b^	1681 (771–2514)^ab^	<0.0001

Plasma levels of TNF-∝, IFN-γ, IL-4, IL-10, IL-12 and TGF-β for 75 samples from symptomatic malaria patients and apparently healthy controls had been previously reported [23] and here are combined with IL-6, sTNFRI and sTNFRII measurements in these same samples and a complete analysis of additional samples. Data are expressed as median (interquartile range) unless stated otherwise and compared across three or four groups with Kruskal-Wallis tests (continuous variables) or χ^2^ tests (proportions); *P* values for this comparison across groups are presented in the right-hand column. When comparisons across groups showed statistical significance, further pairwise comparisons were made using Mann-Whitney tests (continuous variables) or χ^2^ tests (proportions). Values with different superscripts across a row indicate pairwise comparisons with statistical significance at the 5% level. The same value may have more than one superscript. When pairs of values across a row share one superscript (either the only or one of the superscripts associated with them), no statistically significant difference was found.

Levels of cytokines or soluble TNF-α receptors often correlated to each other in vivax malaria patients. For example, concentrations of IL-10 correlated positively with those of IL-12p40 (*r*_s_ = 0.452, *P*<0.0001), IFN-γ (*r*_s_ = 0.361, *P* = 0.001), IL-6 (*r*_s_ = 0.439, *P*<0.0001), sTNFRI (*r*_s_ = 0.449, *P*<0.0001) and sTNFRII (*r*_s_ = 0.395, *P* = 0.001). Levels of sTNFRI, but not those of sTNFRII, correlated negatively with TNF-α concentrations (*r*_s_ = −0.267, *P* = 0.038). Levels of both TNF-α receptors correlated positively with those of IL-6 (*r*_s_ = 0.413 [*P* = 0.001] and *r*_s_ = 0.416 [*P*<0.0001] for sTNFRI and sTNFRII, respectively). Among asymptomatic *P. vivax* carriers, concentrations of IL-10 correlated positively with those of IL-12p40 (*r*_s_ = 0.459, *P* = 0.002), IFN-γ (*r*_s_ = 0.516, *P*<0.001) and sTNFRII (*r*_s_ = 0.429, *P* = 0.003); no correlation was found between TNF-α or IL-6 concentrations and levels of soluble TNF-α receptors.

To address the hypothesis that *P. vivax* elicits greater inflammatory cytokine response per parasitized red blood cell than other human malaria parasites [21], [22], we compared cytokine concentrations adjusted for parasitemias and found these comparisons to be heavily influenced by differences in parasite density. We found significantly higher per-parasite levels of TNF-α in mixed-species symptomatic infections (median density, 228 parasites/µl) than in single-species symptomatic *P. vivax* infections (median density, 1184 parasites/µl). However, the highest per-parasite levels of cytokines and soluble TNF-α receptors were found among asymptomatic carriers of very low *P. vivax* parasitemias (median density, 16 parasites/µl), consistent with a non-linear relation between parasite density and cytokine concentrations in vivax malaria.

Because IL-10 regulates the production and function of inflammatory cytokines [35], we computed IL-10/TNF-α, IL-10/IFN-γ and IL-10/IL-6 ratios and found a bias toward regulatory cytokines in clinical vivax malaria (Table 2). Nevertheless, no clear anti-inflammatory bias could be observed among asymptomatic carriers of *P. vivax,* who typically had low plasma concentrations of both inflammatory and regulatory cytokines (Table 2).

### Cytokines and Soluble TNF-α Receptors in Relation to Clinical and Laboratory Outcomes during *Plasmodium vivax* Infection

The relationships between levels of circulating cytokines during acute malaria episodes (independent variables) and four outcomes (dependent variables): hemoglobin levels, platelet counts, parasitemias, and symptom intensity, were then explored. Among 82 patients with clinical vivax malaria, age (β = 0.167, *P* = 0.005), gender (β = 0.612, *P*<0.001) and parasitemia (β = −0.231, *P* = 0.009), but none of the cytokines or soluble TNF-α receptors, were identified as independent predictors of hemoglobin levels in vivax malaria in multiple linear models. Similar results have been previously described in vivax malaria patients from Brazil [33] and India [36]. The next model incorporated both symptomatic and asymptomatic *P. vivax* infections (complete data for 109 subjects) and yielded quite similar results: age (β = 0.190, *P* = 0.017), gender (β = 0.528, *P*<0.001) and parasitemia (β = −0.184, *P* = 0.021) emerged as the only significant predictors of hemoglobin levels.

Next, we tested whether cytokines were associated with thrombocytopenia, a common complication of *P. vivax* infections in this (Table 1) and other studies [37], [38]. Levels of the following cytokines were negatively correlated with platelet counts on partial linear regression models with data for symptomatic vivax malaria infections: IL-12p40 (β = −0.274, *P* = 0.014), IFN-γ (β = −0.254, *P* = 0.020), IL-10 (β = −0.310, *P* = 0.004), IL-10/TNF-α ratio (β = −0.321, *P* = 0.003) and sTNFRII (β = −0.241, *P* = 0.034). Regression models including IL-12p40, IFN-γ, IL-10 or IL-10/TNF-α ratio and sTNFRII as independent variables, in addition to age, gender, length of exposure to malaria and parasitemia, revealed that parasitemia (β = −0.332, *P* = 0.005) and age (β = −0.419, *P*<0.001), but none of the other variables, were independent predictors of platelet counts in vivax malaria. Only age (β = 0.235, *P* = 0.014) remained as a significant independent predictor of platelet counts in a multiple linear regression model with all *P. vivax-*infected subjects with complete data (n = 109), either symptomatic or not.

Although levels of several cytokines were proportional to parasitemias in symptomatic vivax malaria, the direction of the correlation varied. For instance, IL-4 (β = −0.282, *P* = 0.007) and TGF-β (β = −0.303, *P* = 0.004) were negatively correlated, while IL-10 (β = 0.297, *P* = 0.004) and sTNFRII (β = 0.259, *P* = 0.022) levels and IL-10/TNF-α (β = 0.306, *P* = 0.002), IL-10/IFNγ (β = 0.388, *P*<0.0001) and sTNFRII/TNF-α ratios (β = 0.242, *P* = 0.033) were positively correlated with parasite counts. Final linear regression models that comprised IL-4, TGF-β, IL-10 or IL-10/TNF-α or IL-10/IFNγ ratio and sTNFRII or sTNFRII/TNF-α ratio as independent variables, in addition to age, gender and length of exposure to malaria, pointed to the length of exposure to malaria (β = −0.330, *P* = 0.002), TGF-β levels (β = −0.323, *P* = 0.003), and the IL-10/TNF-α ratio (β = 0.332, *P* = 0.004) as major independent predictors of *P. vivax* parasitemias. Therefore, anti-inflammatory cytokine levels may correlate either positively (IL-10; as reported elsewhere [36], [39], [40]) or negatively (TGF-β) to parasite density in uncomplicated but symptomatic vivax malaria. A positive correlation was also found between sTNFRII levels and parasite density in vivax malaria (β = 0.526, *P* = 0.015), consistent with previous studies of *P. falciparum* infections in Africa [17], [41], [42]. Next we tested whether multiple linear models incorporating asymptomatic carriers of low-level *P. vivax* parasitemia would yield similar results (complete data for 127 subjects). We found that plasma concentrations of sTNFRII (β = 0.361, *P*<0.001) and either IL-10 levels (β = 0.503, *P*<0.001) or the ratios IL-10/TNF-α (β = 0.324, *P* = 0.001) or IL-10/IFNγ (β = 0.476, *P*<0.001) correlated positively with parasite density in human infection with *P. vivax,* regardless of the presence of symptoms.

No evidence was found suggesting that vivax malaria patients tended to report milder clinical symptoms with increasing levels of regulatory cytokines. To the contrary, IL-10 concentrations and the IL-10/TNF-α ratio (β = 0.270, *P* = 0.014 and β = 0.225, *P* = 0.046, respectively), in addition to levels of IL-6 (β = 0.302, *P* = 0.014) and sTNFRII (β = 0.258, *P* = 0.035), were positively correlated with the overall clinical index on partial multiple linear regression models. The overall intensity of symptoms did not correlate with patient age, length of residence in Amazonia, a proxy of cumulative exposure to malaria, or with parasitemia. Multiple regression models with IL-10 or IL-10/TNF-α ratio, IL-6 and sTNFRII failed to detect any independent correlation between these cytokines and the overall clinical index because of the clear co-linearity between many variables.

IL-10, IL-6 and sTNFRII levels (β = 0.308, *P* = 0.005, β = 0.248, *P* = 0.043 and β = 0.273, *P* = 0.023, respectively) and the IL-10/TNF-α ratio (β = 0.225, *P* = 0.046) were also correlated with the intensity of paroxysm-related symptoms on partial regression models. Similarly, multiple linear regression models with IL-10 or IL-10/TNF-α ratio, IL-6 and sTNFRII were affected by co-linearity between variables and failed to detect independent predictors of paroxysm-related symptom intensity. We conclude that a less inflammatory cytokine response in vivax malaria did not translate into milder overall clinical manifestations or less intense paroxysms.

### Cytokines and Soluble TNF-α Receptors After Antimalarial Chemotherapy

To examine whether levels of cytokines and soluble TNF-α receptors remained elevated after parasite clearance following chemotherapy, acute-phase and convalescence samples from *P. vivax* malaria patients given standard chloroquine-primaquine treatment were compared. No malaria parasites were detected by real-time PCR in convalescence samples, and platelet counts had returned to within the normal range after chemotherapy. Plasma levels of IL-12p40, IL-6, IFN-γ, IL-10, TGF-β, sTNFRI and sTNFRII had decreased significantly, while those of IL-4 and TNF-α remained stable during convalescence (Table 3), but were elevated compared to non-infected controls (Table 1). The 40-fold decrease in IL-10 levels in convalescence samples is particularly noteworthy.

**Table 3:** Cytokine levels in acute-phase and convalescence blood samples from *P. vivax*-infected study participants.

Variable	Acute phase	Convalescence	No. of paired samples	*P value*
Hemoglobin (g/ml)	134 (123–149)	135 (123–142)	39	0.850
Platelets (10^9^/ml)	158 (113–206)	235 (213–302)	39	<0.0001
IL-4 (pg/ml)	4.67 (3.0–6.8)	3.9 (3.8–5.6)	36	0.220
IL-12 (pg/ml)	66.5 (49.4–101.7)	39.1 (29.9–55.1)	33	<0.0001
IL-6 (pg/ml)	13.6 (10.2–29.5)	9.6 (9.1–10.8)	39	<0.0001
IFN-γ (pg/ml)	12.5 (5.8–57.5)	7.5 (2.9–12.3)	37	0.011
TNF-α(pg/ml)	7.3 (5.1–10.1)	8.6 (4.0–11.3)	37	0.777
IL-10 (pg/ml)	297.4 (79.9–523.9)	7.2 (4.5–9.7)	37	<0.0001
TGF-β (pg/ml)	712.6 (378–4115)	538 (375–1008)	36	0.011
sTNFRI (pg/ml)	3167 (1821–5798)	610 (542–681)	35	<0.0001
sTNFRII (pg/ml)	15363 (11175–34243)	4269 (2498–5734)	39	<0.0001
IL-10/TNF-α ratio	43.4 (7.4–68.4)	1.0 (0.7–1.5)	37	<0.0001
IL-10/IFN-γ ratio	14.1 (3.6–41.4)	1.9 (0.7–1.9)	37	<0.0001
IL-10/IL-6 ratio	11.2 (3.0–28.3)	0.6 (0.4–0.9)	33	<0.0001
sTNFRI/TNF ratio	405.3 (206.7–872.2)	116.4 (58.1–172.6)	29	<0.0001
sTNFRII/TNF ratio	2930.9 (1323.3–4436.4)	601.4 (263.5–1298.7)	33	<0.0001

Levels of TNF-∝, IFN-γ, IL-4, IL-10, IL-12 and TGF-β in 22 paired samples had been reported previously [23] and were combined here with those for 17 additional pairs. Data are expressed as median (interquartile range) and were compared using Wilcoxon tests.

## Discussion

In this study, we confirm early findings [23] of contrasting patterns of cytokine balance in uncomplicated single-species symptomatic infections with *P. vivax* and *P. falciparum* and in co-infections with both species diagnosed in the same area of Brazil. Contrary to the recent suggestion that *P. vivax* elicits more inflammation than *P. falciparum* [21], [22], we found higher IL-10/TNF-α, IL-10/IFN-γ and IL-10/IL-6 ratios, but similar inflammatory cytokine responses per parasitized red blood cell, in vivax compared to falciparum malaria. IL-10/TNF-α ratio and sTNFRII levels correlated positively with *P. vivax* parasitemia, but not with symptom intensity. Furthermore, we showed that the predominantly anti-inflammatory cytokine response in clinical vivax malaria was short-lived, with IL-10 concentrations and IL-10/TNF-α, IL-10/IFN-γ and IL-10/IL-6 ratios all returning to within the normal range found in non-infected controls after chemotherapy.

Asymptomatic carriers of very low *P. vivax* parasitemias living in the same region had substantially lower responses of both inflammatory and regulatory cytokines than did patients with clinical vivax malaria. They had higher levels of IL-4, IL-10 and IL-12p40 than non-infected controls. Interestingly, asymptomatic parasite carriers had the highest median IL-4 levels among all groups of study subjects, either infected or not. Nevertheless, their median IL-10 concentrations were more than 25-fold lower than those found in symptomatic *P. vivax* infections. Concentrations of sTNFRI and sTNFRII among asymptomatic carriers of *P. vivax* were similar to those found among non-infected controls and 3 to 4-fold lower than those found in symptomatic *P. vivax* infections. The finding of relatively low levels of most cytokines in asymptomatic carriers of low-grade parasitemias suggests that a parasite-density threshold must be reached before substantial cytokine responses are triggered. In addition, we found no evidence for an anti-inflammatory bias in the cytokine responses of asymptomatic carriers of *P. vivax.*

We and others [33], [34], [40] have shown prominent IL-10 responses in symptomatic but uncomplicated *P. vivax* malaria, but their main cellular sources remain largely unknown. Adaptive type 1 regulatory (Tr1) CD4^+^ cells, which do not express CD25, FoxP3 or CD127 on their surface, have recently been identified as the main source of IL-10 in experimental murine infection with *P. yoelii* [35]. However, in *P. chabaudi* infections in mice, activated effector T_H_1 cells (IFN-γ^+^, ICOS^high^, and CD127^high^) are major producers of IL-10 [43]. Understanding how these non-classical regulatory cells are induced could help to explain species-related differences in IL-10 production in human malaria.

Our cytokine measurements at a single time point are unable to provide insights into the sequence of events from parasite inoculation through the onset of clinical disease [11]. The direction of some putative causal relationships is hard to infer. For example, the positive correlation between IL-10 levels and *P. vivax* density [39] and the detection of low plasma concentrations of IL-10 in asymptomatic carriers of very low parasitemias (Table 2) allow for two competing interpretations. High IL-10 levels may be a consequence of high parasite density if a minimum density threshold is required to trigger substantial IL-10 production. Alternatively, increased IL-10 levels may favor parasite multiplication by inhibiting parasite-killing effector mechanisms in humans [44] and mice [35].

Similarly, the findings of high levels of IL-4 among asymptomatic carriers of low parasite loads and a negative correlation between IL-4 levels and parasitemias could be interpreted as indicative that a strong IL-4 response helps to control *P. vivax* parasitemias (and thus reduce the risk of malaria-related disease) at the early stages of infection. Nevertheless, a single IL-4 measurement during the course of infection does not allow us to infer a causal relationship; alternatively, we may have simply detected higher IL-4 levels in subjects that were enrolled at the early stages of infection, while parasite densities are still low and disease is less likely to occur.

The sharp increase in TNF-α levels preceding febrile paroxysms in vivax malaria [45] can elicit the release of soluble TNF-α receptors, which in turn regulate TNF-α activity. At low concentrations, soluble TNF-α receptors, which are derived from proteolytic cleavage of the cell-surface TNF-α receptor, stabilize TNF-α and prolong its half-life [46]. At high concentrations, however, they compete with TNF-α for binding with cellular receptors, particularly membrane-bound TNF-α [47], thereby inhibiting TNF-α bioactivity [48]. As a consequence, soluble TNF-α receptors may be a more reliable biomarker of parasite-induced inflammation than short-lived TNF-α and other classical pro-inflammatory cytokines [49]. Since our vivax malaria patients have higher sTNFRII concentrations (which correlate positively with parasitemia) and sTNFRII/TNF-α ratios than falciparum malaria patients, they have comparatively less free TNF-α available for interaction with cell surface receptors.

Cytokine responses reflect different host strategies for controlling infection with different malaria species [50]. Controlling virulent, fast-multiplying parasites such as *P. falciparum* blood stages may require a double-edged strategy: a strong inflammatory response that can prevent fulminant infections but which may lead to severe disease. We hypothesize that tight control of parasite growth is not required in vivax malaria, since parasites find a limited supply of reticulocytes to parasitize in peripheral blood and there is no risk of overwhelming parasitemias. As a consequence, preventing inflammation-related tissue damage with an early regulatory cytokine response may be the top priority, although this strategy favors some parasite multiplication. Recent advances in our understanding of the pathophysiology [4]–[6] and immunopathology [24], [51], [52] of severe vivax malaria provide novel bases for interpreting cytokine responses and their consequences in this major human infection.

## Supporting Information

File S1 **Methods online.** Detailed description of malaria diagnosis by conventional microscopy, molecular diagnosis of malaria and antimalarial treatment.
(DOC)
