# Supplementary material for: Cytokine Balance in Human Malaria: Does Plasmodium vivax Elicit More Inflammatory Responses than Plasmodium falciparum?
Source: PLoS One. 2012 Sep 4;7(9):e44394. doi: 10.1371/journal.pone.0044394 (PMC3433413; doi:10.1371/journal.pone.0044394)
Supplement: File S1 — Methods online. Detailed description of malaria diagnosis by conventional microscopy, molecular diagnosis of malaria and antimalarial treatment. (DOC) [file pone.0044394.s001.doc]

#### S1: Supplementary Methods online

#### Malaria diagnosis by conventional microscopy

#### For microscopical diagnosis of malaria, thick blood smears were stained with Giemsa and at least 100 fields were examined for malaria parasites under 1000 × magnification. On average, each microscopist has examined an average of 0.1 µl of blood. Under field conditions, the average detection threshold of conventional microscopy is around 84 parasites per µl of blood [1], which would theoretically correspond to 8.4 parasites in 100 high-power fields. However, up to 20% of parasites are usually lost during thick-smear staining [2], reducing considerably the sensitivity of conventional microscopy.

#### Molecular diagnosis of malaria

#### We standardized a quantitative real-time PCR that targets the *18S rRNA* gene to quantify *P. vivax* and *P. falciparum* parasitemias in all clinical samples. DNA templates for PCR amplification were isolated from 200 μL of whole venous blood using QIAamp DNA blood kits (Qiagen, Hilden, Germany). Each 20 µL reaction mixture contained 2 µL of sample DNA, 10 µL of 2× Maxima SYBR Green qPCR master mixture (Fermentas, Vilnius, Lithuania) and 0.5 μM of each primer. We used the genus-specific primer P1 (ACG ATC AGA TAC CGT CGT AAT CTT) combined with either of the species-specific primers, V1 (CAA TCT AAG AAT AAA CTC CGA AGA GAA A) or F2 (CAA TCT AAA AGT CAC CTC GAA AGA TG), for *P. vivax* and *P. falciparum,* respectively. These primers allow the amplification of a species-specific ~100 bp fragment of the *18S rRNA* gene [3]. Standard curves were prepared with serial tenfold dilutions of the target sequence, cloned into pGEM-T Easy vectors (Promega, Madison, WI), to allow for species-specific quantitation of parasite loads (number of parasites/μL of blood). For subjects co-infected with *P. vivax* and *P. falciparum,* parasite counts for each species were summed to calculate the total parasitemia. We used a Mastercycler Realplex S real-time thermal cycler (Eppendorf, Hamburg, Germany) for PCR amplification with an initial step at 50 ° C (2 min), template denaturation at 95° C (10 min), followed by 40 cycles of 15 s at 95° C and 1 minute at 60° C, with fluorescence acquisition at the end of each extension step. Amplification was immediately followed by a melting program consisting of 15 seconds at 95° C, 15 seconds at 60° C, and a stepwise temperature increase of 0.03° C/s until 95° C, with fluorescence acquisition at each temperature transition.

#### The estimates of parasite density obtained by quantitative real-time PCR are not directly comparable to those obtained by conventional microscopy for several reasons. First, the proportion of parasites or parasite DNA that is lost during sample processing (thick smear staining or DNA extraction) varies widely according to the diagnostic method used. Second, the number of copies of the target sequence (the *18S rRNA* gene) may vary in the genome of malaria parasites. In our analysis we have assumed that each genome of *P. falciparum* and *P. vivax* has five copies of the *18S rRNA* gene (as found in the 3D7 genome), but up to eight copies have already been estimated to occur in the genome of the K1 strain of *P. falciparum* [4]*.* Such an intraspecific variation may lead to under- or over-estimates in parasite density by a factor of 2, which certainly does not affect our main analysis in this paper but may give a false impression of a poor performance of conventional microscopy when real-time PCR-derived parasitemias are over-estimated. No data on *18S rRNA* gene copy number are currently available for *P. vivax* [4].

## Antimalarial treatment

## Plasmodium vivax episodes were treated with chloroquine (25 mg/kg of base over three days; adult dose, 1.5 g over three days) plus primaquine (0.5 mg/kg/day of base for seven days; adult dose, 30 mg/day); while P. falciparum episodes were treated with mefloquine (15 mg/kg of base, single dose; adult dose, 1000 mg) or artemether (2-4 mg/kg/day; adult dose, 160 mg/day) plus lumefantrine (12-24 mg/kg/day; adult dose, 960 mg/day) over three days. There is no evidence of chloroquine resistance (defined as parasite recrudescence observed despite therapeutic whole-blood levels of chloroquine) in P. vivax populations from this region [5].

**References**

1. Craig MR, Sharp BL (1997) Comparative evaluation of four techniques for the diagnosis of *Plasmodium falciparum* infections. Trans. R. Soc. Trop. Med. Hyg. 91: 279-82.

2. Hommel M (2002) Diagnostic Methods in Malaria. In: Warrell DA, Gilles HM (eds.), *Essential Malariology,* 4^th^ edition. London, Arnold, p. 35-58.

3.Kimura M, Kaneko O, Liu Q, Zhou M, Kawamoto F, et al. (1997) Identification of the four species of human malaria parasites by nested PCR that targets variant sequences in the small subunit rRNA gene. Parasitol. Int. 46: 91-5.

4. Mercereau-Puijalon O, Barale JC, Bischoff E. (2002) Three multigene families in *Plasmodium* parasites: facts and questions. Int. J. Parasitol. 32: 1323-44

5. Orjuela-Sánchez P, da Silva NS, da Silva-Nunes M, Ferreira MU (2009) Parasitemia recurrences and population dynamics of *Plasmodium vivax* polymorphisms in rural Amazonia. Am. J. Trop. Med. Hyg. 81: 961-68
